# A Manual of Medical Jurisprudence, for Physicians and Jurists

**Published:** 1843-07

**Authors:** 


### ART. IV.

*Handbuch der gerichtlichen Medicin, &c. für Aerzte und Criminalisten.* Von Dr. G. H. NICOLAI.—*Berlin,* 1841, pp. 556.

*A Manual of Medical Jurisprudence, for Physicians and Jurists.* By Dr. G. H. Nicolai.—*Berlin,* 1841.

THE volume of Dr. Nicolai professes to be a manual for the guidance of medical men and lawyers in the examination of all medico-legal investigations. The subjects are treated in a somewhat condensed form, but nevertheless we are inclined to regard this work as a good addition to this branch of medical literature. In the first part the author treats of the questions connected with pregnancy, delivery, and criminal abortion. The peculiarities of the fœtus at all stages of gestation are then fully described, more minutely than appears to us necessary, in a work especially intended for practice. Thus we cannot see the necessity for a medical jurist being informed of the exact measurements in length and breadth of all the bones of the child; for it is not by such trivial and uncertain characters as these that he would proceed to determine the age of a child in a case of abortion or child murder. (p. 48.) The pages devoted to these details, might have been well spared for some other subjects of practical importance. The causes of the death of the child before, during, and after birth (p. 54) are very well described; but in other respects, the questions relative to frequency and parturition do not call for particular notice.

The subject of infanticide is somewhat briefly treated, at least as far as concerns that *vexata questio,* the evidence of the child having lived after birth, from the state of its lungs. It is well known that lungs may have undergone the process of respiration, and yet sink in water.

"Lungs will not float in water, when only a small part of their structure has been filled with air, when they are incompletely developed, or only expansible in certain insulated portions. It is a well-known fact that children may live and breathe a short time, provided only a very small portion of the lungs receives air; and they have been known to cry several times, when, after death it has been found that only a few detached parts of the lungs had undergone respiration. The lungs of children which are immature, are very likely to be found in this condition, since these subjects may commence but have not the power to continue the process of respiration, or to give to the chest its full degree of expansion. This state is what has been termed *atelectases* by Jorg. Two cases have occurred to me in which the lungs of one side of the chest floated, and those taken from the other side sunk. These latter were of a dark-red colour, resembling the liver in consistency ; they were congested with blood, and on compressing them, a bloody purulent matter oozed out. Both children had lived, the one two days, the other three weeks." (p. 80.)

The author states upon the authority of Mende, that in children which have survived birth, but have subsequently died from cold, the lungs lose their buoyancy in water. This statement appears to us to require confirmation. Nothing is added to our knowledge on the subject of artificial inflation ; but Dr. Nicolai joins most medical jurists in the opinion, that the operation of inflating the lungs is of too difficult a nature to allow this to be made a serious objection to the employment of the hydrostatic test in cases of infanticide. He contends further, that there is this difference between respired and inflated lungs, that where the air has been received by respiration, " the lungs contain more blood ; on cutting them, the blood streams out abundantly, and the pulmonary artery with its branches is filled with dark-coloured blood. On the other hand, when the lungs have received air by artificial inflation, they appear like a distended bladder or membrane without blood." (p. 81.)

Desirable as it would be in these cases, to find some good physical distinction between respired and inflated lungs, we must decidedly object to the reception of any such criterion as that here laid down by Dr. Nicolai. In the repeated examination of lungs under both conditions, we have not met with this difference which he describes ; and we believe that it would be most unsafe to rely upon it as any evidence of the origin of the air contained in the pulmonary cells. Admitting that the lungs of children after respiration are more congested with blood than before the establishment of that process, many observers have met with cases of congested lungs in still-born children, that had not breathed. The notion that the presence or absence of blood in the pulmonary vessels can assist a medical practitioner in this important inquiry is now very properly exploded. All we can say is, that while Dr. Nicolai's conclusions are theoretically true, they are practically erroneous ; and we cannot, therefore, agree with him in the assertion that a safe reliance may be placed on the quantity of blood in the lungs, to distinguish inflation from respiration.

The author next enters into a review of the various other tests for determining the question of respiration in new-born children. These rather belong to the history of the science, and therefore do not here require to be adverted to. The tests of Ploucquet and Daniel he considers unsafe.

The conclusions of the author on the subject of the hydrostatic and other tests, indicate the degree of uncertainty which attends the employment of them. Thus the buoyancy of the lungs in water is not a decided proof of respiration, nor is there absolute weight, unless taken with other circumstances. When the signs of respiration are entirely wanting, it cannot be affirmed with certainty, that the child has not respired. Respiration may have been performed, but imperfectly, and no physical signs of the process remain in the lungs. It is well known that a child may live some time after its birth without respiring ; but there is not one of the tests which can establish this condition. The sinking of the lungs and the absence of the other phenomena of respiration do not show that the child died *before* birth, and lastly, the fact whether the child breathed *before*, *during*, or *after* its birth, cannot be determined by these tests under any circumstances whatever. (p. 94.)

We fully agree with him, that because these difficult points cannot be settled by the hydrostatic test, this is no objection to its employment in cases of infanticide. Questions of this nature are entirely beyond the reach of its application ; and it would be just as reasonable to reject the tests for arsenic, because they are unfitted to detect the presence of corrosive sublimate, as to reject this test on the ground above alluded to.

In dismissing this part of the subject we must here call the attention of English medical jurists to a point which is becoming of considerable importance, in relation to the medical evidence given in cases of child-murder. The difficulty in the present day, is not so much to determine whether or not a child has breathed, as to establish whether the act of respiration was performed *before* or *after* the *entire birth* of its body. It will be observed that at every assizes, where a charge of infanticide is tried, and such cases are now very numerous, the medical evidence may be clear as to the proof of respiration ; but unless the witness is prepared to swear that the process was set up or continued *after* the body of the child was entirely in the world, this evidence is not held to be sufficient. The judge commonly stops the case by remarking that the proof of live birth has failed. We fully concur in the opinion of Dr. Nicolai, that in the present state of science, there is no certain sign whatever to indicate where the act of respiration took place in children that have lived but a short time. It is beyond the reach of any medical evidence to show whether the act was performed during the protrusion of the head from the outlet, or after the entire delivery of its body. There can obviously be no difference in the state of the lungs ; for we are supposing, as it happens in most cases of alleged childmurder, that the child lives but a very short time. It is, therefore, improper to charge the hydrostatic test with a deficiency which cannot be fairly imputed to it. The point insisted upon as a necessary part of the evidence by our judges, requires some new proof or test which at present it appears to be beyond the reach of science to afford. In the mean time, the definition of child-murder should be changed ; it is not the wilful destruction of a living child which the evidence is required to prove, but the destruction of a child which is proved to have breathed after every portion of its body was in the world. In the absence of ocular evidence, no proof of this kind can be possibly afforded ; and as the benefit of the presumption is always given to the prisoner, so we do not see how with an advocate who thoroughly understands the difficulties of the subject, any prisoner, however guilty of the death, can in future be convicted of the crime of destroying her offspring.

The characters of infancy and puberty are next set forth ; and we must here particularly point out the account of the changes that take place in the skeleton of the child. These are very well detailed, and they are well adapted to assist the practitioner in determining the age of a skeleton that may have been discovered buried under suspicious circumstances.

There are many questions connected with the bones of the adult after various periods of interment, which medical practitioners often find considerable difficulty in answering. This is owing to the subject of osteology never having been considered by them in its relations to legal medicine.

"Injuries to bones produced some time before death are known by the softer consistency and enlarged appearance of the parts involved. Traces of callus may also be found where there has been fracture. In determining for how long a period bones may have remained interred in the ground, it is necessary to consider the age of the individual; for this materially affects the rapidity of decomposition, as well as the kind of soil in which they have been interred. The bones of subjects abounding in liquid matter, of young persons and children, undergo decay with greater rapidity than those of old and emaciated subjects. The firm and hard bones resist decomposition longer than those which are soft and cartilaginous. Soils formed of loam or compact clay retard these changes in the bones: but in light sandy or calcareous soils decomposition is accelerated. The more freely bones are exposed to air, the more readily do they become decomposed. In general, the soft putrefaction of bone ceases in about twenty or thirty years from the time of interment. After this period, all the soft parts are destroyed: they are deprived of gelatin, and will be found hard and porous. The cartilages disappeared with the marrow and the fatty parts. If in examining bones, any of the soft parts remain, if they are oily and moist, and still contain within them some marrow, it may be inferred that they have lain within the earth from ten to fifteen years. If the bone readily crumbles under pressure; is rough and porous throughout its whole substance, it may be presumed that it has been in the ground forty, fifty, or even one hundred years." (p. 174.)

Although Dr. Nicolai's observations may be generally true, yet it must be remembered that there is a great dearth of facts in relation to the changes of bone within a given period. We have found gelatin and oil in bones that had been interred in chalk for a period of thirty-four years; and animal matter in bones that had lain in loose clay for upwards of half a century. Indeed it is a well-known fact, that bones may retain a portion of their animal matter, even after an interment of three or four centuries. There are so many influences that affect the rapidity of decomposition in bone, that it is difficult to lay down any general rules for determining the probable period of interment in an unknown case. Each case must be judged of by itself; at any rate we must be careful that we do not pronounce for a short period of interment, merely from the fact that there are still traces of animal matter in the substance of the bone.

The author entirely passes over one important question connected with the exhumation of bones, namely the mode of distinguishing the sex. There are many cases on record, where this evidence has been material, and it is easy to perceive that this is a question of as much importance as that which relates to the period of interment. In the early periods of life the means of distinguishing the bones of the sexes are very imperfect, and in consequence mistakes have frequently been made. Even in the adult subject, unless the whole of the skeleton has been disinterred, there may be some difficulty in pronouncing a positive opinion. The pelvis is well known to furnish the best mark of distinction between the skeletons of the two sexes; but the medical practitioner may merely have some of the bones of the upper or lower extremity, or a few of the ribs to guide his judgment. In such a case a decision will be difficult. The bones of the male are commonly stouter, heavier, and longer than those of the female; and the processes and ridges are more strongly marked upon them, but it is undoubted that there are many male skeletons in which these characters are not very apparent; and other female skeletons the characters of which closely resemble those of the male.

The section on wounds does not satisfy us. The author has followed that bad artificial arrangement, which is adopted by most of his countrymen, and which takes its character from the singular state of jurisprudence existing in Germany in relation to local injuries. Thus we have absolutely mortal, conditionally mortal, and collaterally mortal wounds! although the injuries themselves afford no ground for such an arbitrary arrangement. This system of judging of the criminality of prisoners, not according to the intention displayed by the act of wounding, or the obvious results of their violence, but according to certain arbitrary rules serves no other purpose than to perplex medical witnesses, and to interfere with the proper administration of justice. Thus if a man is stabbed by an assailant, and he dies from hemorrhage, it can signify but little in relation to the wrong committed, whether the wound is in the brachial or in the common carotid artery; and yet according to this system of jurisprudence, there is a difference in the complexion of the offence; the one being a conditionally, and the other an absolutely mortal wound. In England the main inquiry would be whether death was caused by the wound; the prisoner would not derive any benefit from its anatomical situation, nor would it be necessary to speculate upon whether, had a surgeon happened to have been present, the deceased might have been saved in one case, although not in the other. Cases are of daily occurrence which show the absurdity of laying down or acting upon such capricious rules in relation to the mortality of wounds. The whole of this section is, in a practical view, without any interest to the English medical jurist; for the facts are stated by the author simply with a reference to the practice of the German courts. There is also an entire want of illustrative cases; and we know of no department of the science where teaching by examples could be made so profitable as in this. There are several faults of omission. Thus the subject of ecchymosis, involving numerous and highly important medico-legal questions, is entirely overlooked; nor do we find any rules mentioned for distinguishing between wounds inflicted on the living and the dead; and yet there is no doubt that as a matter of practice, this is a question of far greater moment than the settlement of the relative degrees of mortality in wounds of different parts of the body.

The subject of *malapraxis* in relation to the various branches of medicine has been but little examined by English medical jurists; although it must be admitted to be one of great interest to the profession. Incompetency in a regular, or ignorance in an irregular practitioner, appears to become a frequent matter of public investigation in Germany; but it seldom extends beyond a coroner's inquest in England. When trials for malapraxis have occurred, they have been in general ill reported, so that it was not easy to find out the truth of the case, or to make a profitable use of the report. Indeed such cases are confined to the daily journals: they are seldom met with in medical periodicals, and thus they are lost to the profession for all the purposes of medico-legal practice. The author treats concisely of malapraxis in relation to surgeons, accoucheurs, and apothecaries; but it is easy to see that his attention has been principally given to obstetric practice; and it is well known that more faults are here committed by irregular or ignorant practitioners, than in any other department of medicine. The victim of mistreatment is sent to the grave, and nothing further is heard of the case. The only remedy for such a state of things must be a rigorous examination, and a special licence to practise. The malapraxis of chemists, or rather of dispensing druggists, is a matter which should likewise receive the attention of the legislature. It must be a matter of astonishment to all who reflect on this subject, that an individual should be allowed to dispense medicine without having given any test of his capacity to undertake so responsible an office; and yet this is at present the anomalous state of medical government in England. In this respect we form an exception to all other countries in Europe.

Dr. Nicolai devotes a long chapter to feigned and simulated diseases; but we do not consider this a subject involving many practical difficulties. Medical men are not often consulted in such matters in civil life; and when they are, they frequently do not show greater aptitude in discovering tricks and impositions than the civil authorities. This chapter is more especially adapted for army and navy surgeons, or those who have the medical superintendence of prisons.

In the chapter on suicide, we find the following remarks on the means of determining the period at which a gun or pistol has been discharged. The time may be divided into four periods:

"The first period lasts about two hours, and is known by the dark blue colour or efflorescence left on the piece by the discharge of the powder, by the absence of crystals of red oxide of iron (?) or of any salt of iron: by the slight turbidness of the solution derived from washing the piece, and the presence of sulphuretted hydrogen gas.

"Ferrocyanate of potash, (?) acetate of lead and tincture of galls will show whether sulphuretted hydrogen gas be present or not. As additional tests, we may observe in the first instance, the absence of any salt of iron, afterwards its presence, and finally its partial disappearance.

"The second period lasts twenty-four hours. The efflorescence on the weapon is not of so deep a colour: the solution is clear, there is no trace of sulphuretted hydrogen, or of red oxide of iron, and some salt of iron is present in the liquid.

"The third period lasts thirty days. Small crystals appear about the pan and touchhole; and these are long in proportion to the length of time since the discharge. Many traces of red oxide of iron may be found about the weapon, the presence of which may be easily shown by using the tincture of galls and the ferrocyanate of potash.

"The fourth period lasts about fifty days, and is distinguished from the third by there being less of the salt of iron, and more of the red oxide of that metal." (p. 293.)

Imperfect as this method of determining the period of the discharge of fire-arms must appear, we have quoted it because it displays a novel kind of research, and because we believe it to embrace all that is known relative to the subject. We do not think evidence of this kind would be received without opposition in an English court of law, but the practitioner should be aware of the attempts that have been made by continental medical jurists to furnish answers to these intricate inquiries. No one can doubt that great importance must often be attached to the inquiry as to the probable period at which a particular weapon was discharged: for it may make all the difference between murder and suicide; but many may doubt the propriety of basing the answer upon such loose statements as these above given. In the first instance, the saline residue on the piece consists almost exclusively of sulphuret of potassium. It is alkaline, and is immediately affected by a salt of lead. In the course of from half an hour to an hour, we have found this sulphuret transformed into sulphate, where the air had free access to the weapon; and the solution lost its alkalinity and no longer affected a salt of lead. We never found any traces of iron where the discharge had taken place several hours previously. All we are entitled to say is, that the presence of an alkaline sulphuret indicates a recent discharge; but it is, we believe, impossible to specify the exact time at which it actually took place. In speaking of death from lightning the author observes:

"I have met with cases where a large portion of the surface of the body had been burnt by the action of the electric fluid, and the skin from the head downwards to the lower part of the trunk had become completely excoriated. The clothes were torn in the same direction, and in some parts were burnt or singed. The healing of these wounds was as difficult and painful as in cases of severe burns." (p. 315.)

It is the opinion of medical jurists, that the electric fluid does not produce burns on the body, unless some part of the clothing be ignited; and then of course the burning is an indirect result. We are inclined to think that this was the origin of the burns and excoriations in the instances referred to by Dr. Nicolai. In cases that have been carefully observed, where the dress of the individual has escaped combustion, the wounds produced by the electric fluid which had penetrated beyond the surface presented merely the characters of lacerated punctures, exactly resembling stabs with a blunt dagger.

The author is inclined to admit the doctrine of *spontaneous combustion* in the human body; but as he adduces no new cases to show that this alleged phenomenon ever began in the interior of the body, or even on the exterior without the contact or vicinity of fire, we do not see how the occurrence of this condition is placed, as he asserts it is, beyond the possibility of doubt. We are surprised that he should not have seen the necessity for better evidence to support his opinion than that which he adduces. He admits that all of these subjects have not been spirit drinkers; consequently a separate hypothesis must be invented to account for the spontaneous combustion of those whose bodies have not been impregnated with alcohol. Whatever opinion may be adopted, the subject has very little interest for the medical jurist; since the marks of design or accident in burning are commonly so clearly distinguishable, that not even a medical or scientific opinion is required, to satisfy a court of justice of the real mode of death.

The examination of blood-stains found on the dress of suspected persons has of late become an important part of the duty of a medical jurist. Dr. Nicolai does not attach much importance to inquiries of this sort, if we are to judge by the few lines in which the subject is treated:

"'A very simple method of recognizing suspected blood-stains in medico-legal investigations, is, besides the observation of the colouring particles, to examine them by the light of a candle, in which case the slightest spots will appear of a clear red colour.

"Marks of blood on carpets, wood, or furniture generally, are not easily discovered by the light of day, but by the use of a candle, either at night or in the day time, spots no larger than half a line in diameter (1-24th of an inch) may be easily detected; after this they may be removed and examined microscopically or chemically. When dissolved in water, the red particles may be readily perceived." (?) (p. 321.)

This is all that the author says on the subject.* The methods for distinguishing other stains resembling those of blood, and easily to be confounded with them, are omitted, although this is assuredly a matter highly important in a practical view. There is no objection to the use of the lighted candle in the manner suggested; but a chemical analysis of the suspected stain should never be neglected. The best characters to distinguish blood are the ready solubility of the stain in water, the entire destruction of the red colour by coagulation on boiling, and the fact of the red solution not being turned to a crimson or green colour on the addition of a few drops of ammonia.

The subject of *insanity* is treated at considerable length; but as the points to which the author refers his readers chiefly relate to the rules and ordinances of Prussian law, they are without interest to an English practitioner. The medical history of the subject presents no novelty.

The last section of the work is devoted to *toxicology*. Dr. Nicolai defines a poison to be a substance which, when taken in small quantity, produces dangerous effects upon the system. This we know is a very common definition of a poison; but in our view it is erroneous and highly objectionable in its application to legal medicine. It is clear that if this definition were correct, the class of poisons would embrace but very few substances. Oxalic acid is a powerful poison, and yet compared with arsenic, it requires to be taken in very large doses in order to produce fatal effects. It cannot be doubted that carbonate of lead and tartarized antimony are poisons, but, compared with strychnia it requires a very large dose of either substance to affect the body dangerously or to cause death. Barristers, in defending prisoners on charges of poisoning, have taken advantage of the error in this definition, and have contended that no conviction could take place upon such a charge, because the substance administered was not a poison according to this usual medical definition. We repeat then, that a medical jurist must look to the effects of the substance upon the body, to characterize a poison: the term cannot be with any propriety restricted to that class of substances only which operate powerfully in *small* doses.

Dr. Nicolai has treated very fully the subject of arsenical poisoning, and the chemical processes for the detection of arsenic with the recent improvements are very well described. In his preface, the author acknowledges himself to be indebted to Dr. F. Simon, for an account of the chemical analysis of poisons, and we may observe that this gentleman has given a very able summary of what is at present known on the subject. While the chemical processes are often more simple, the action

* There is a paper inserted as an appendix at the end of the volume, which, however, appears to be a literal translation of Lassaigne's essay on the subject published some years since in the Révue Médicale.

of the tests is more clearly given than in most French and English works on medical jurisprudence.

As we might have expected, much space is given to an account of Marsh's test, and to the cautions which ought to be observed in its employment. The purity of the zinc and sulphuric acid having been determined by previous experiment, the operator must take care not to employ the same zinc a second time; for as the author observes, a portion of arsenic is liable to be precipitated on the zinc, which cannot be removed from it by merely washing with acid water. It is worthy of the attention of those who employ this test, that the arsenieal crust may be deposited, but subsequently volatilized by holding the plate of glass or porcelain too long within the flame. Thus, should the quantity of arsenic be small, it may be erroneously supposed that none was present. The brightest and most metallic-looking stains, are obtained by merely depressing the point of the flame with the glass plate. It is recommended, however, when the quantity of arsenic is minute not to burn the gas; but to pass it through a small glass tube cemented into the apparatus, and to apply a strong heat by a spirit-lamp to one part of this tube. When the glass is heated nearly to redness, the gas is decomposed, the hydrogen escapes, and the metallic arsenic is deposited in a ring at a short distance from the heated spot. (p. 465.)

In distinguishing the antimonial from the arsenical deposit, the author refers to a difference which we have described in a former Number in reviewing Orfila's researches, namely, that when the crust is thin, the stain of arsenic is brown, that of antimony is of a dull gray or smoky black colour. There is, however, a new character which, he says, was first pointed out by Bischoff, namely, that the arsenical stains are all soluble in a solution of chloride of soda, (Labarraque's bleaching liquid,) the thin stain immediately, the black deposits more slowly. In decomposing this solution by muriatic acid, and gently warming it to expel chlorine, we obtain a liquid from which sesquisulphuret of arsenic is easily thrown down on passing into it a stream of sulphuretted hydrogen gas. The stains produced by antimony from Marsh's apparatus are wholly unaffected by a solution of chloride of soda. (p. 466.)

Supposing this to be confirmed by further observations, chemists will have here a good practical ground of distinction, between arsenic and antimony, as well as an easy method of separation when they happen to be deposited in a mixed state upon glass. Dr. Simon does not appear to have met with an account of the simple plan for distinguishing the stains lately proposed by Marsh himself, namely, to burn the gas, and allow the solid product of combustion to fall upon a few drops of ammonio-nitrate of silver in a saucer. If the flame contain arsenic, yellow arsenite of silver is formed; if antimony, silver is reduced and there is a black stain. It has been lately attempted to impugn the value of this mode of testing by the assertion, that if a phosphuret were present in the apparatus yellow phosphate of silver would be formed, resembling the arsenite in colour, and thus a phosphuret would be mistaken for arsenic. This is an objection more ingenious than sound. Mr. Marsh's correcting test answers the purpose intended, it clearly distinguishes arsenic from antimony: and supposing that an alkaline phosphuret finds its way into a liquid for analysis, although we do not see how this can easily happen, it requires no great chemical skill to distinguish such a substance from arsenic. At any rate one who was unable to make this distinction could not safely be trusted with the use of Marsh's apparatus under any circumstances. We do not wish to underrate the difficulties attendant upon the use of this test. They are many: but we object to this wholesale introduction of speculative objections, many of which could only have been concocted in a closet.

Dr. Simon occupies some space in detailing the means for separating arsenic from antimony. (p. 468.) It is well-known that the hydrosulphuret of ammonia precipitates antimony but not arsenic. When the sesquisulphurets require to be distinguished, the better way is to boil the powder in a few drops of strong muriatic acid. The sulphuret of antimony entirely dissolves with evolution of sulphuretted hydrogen, and forms a colourless solution, which, provided the sulphuretted hydrogen be entirely expelled, gives a white subchloride on adding it to a large quantity of water. Sesquisulphuret of arsenic is insoluble in muriatic acid.

The researches of Orfila and Devergie on the detection of arsenic, absorbed into the body, and the experiments of the former on the presence of normal arsenic in the bones and muscles, as a natural constituent, are next adverted to, but Dr. Simon is not aware that Orfila has made a recantation of these views, and that it is now admitted there must have been some mistake in his processes. Arsenic is not present as a natural constituent in any part of the human body. It is unfortunate that Dr. Nicolai's work is thus made a means of diffusing a serious error; but it is a clear proof that medico-legal inferences of so extraordinary a character should be very slowly adopted, and only after a full examination of the facts by many experimentalists. It happened in this case that Orfila stood alone; but his authority was such as to lead readily to the diffusion of a statement as an ascertained fact, before there had been time for others to examine into its correctness, by a repetition of his experiments.

Poisoning by corrosive sublimate is very briefly treated: the pathological effects of this poison are dismissed in a few lines. The chemical analysis is more full: it goes to the detection of mercury only in organic mixtures, by the usual process of immersing in the liquid a slip of gold, having round it a coil of zinc. Dr. Simon condemns the use of tin recommended by Devergie; since, he observes, this often contains mercury, the presence of which, if unsuspected, might lead to a serious error. (483.) Whether this be the case or not, it is certain that the use of gold with zinc is more effectual than that of tin. For the detection of antimony in tartar emetic, sulphuretted hydrogen is recommended as the best test; but the following important fallacy is pointed out by the author:

"The presence of organic substances renders the reaction of this test occasionally obscure. I have remarked that a solution of tartar emetic, mixed with albumen, is not precipitated of a brick-red colour by sulphuretted hydrogen, but of a colour closely resembling the sesquisulphuret of arsenic! The ambiguity is removed by the addition of a few drops of hydrosulphuret of ammonia, which produce the usual orange-red colour." (p. 506.)

In testing for antimony, it must be remembered that the same reagents act very differently on the two principal poisons of that metal, namely, tartarized antimony and chloride of antimony.

"Caustic potash gives with the first a faint turbidness, with the second an abundant white precipitate. Nitric and muriatic acids do not affect the solution of chloride: but they produce white precipitates in the solution of tartar emetic. Ferrocyanate of potash does not precipitate the latter, but it throws down the former, white. Both yield an orange-red precipitate with sulphuretted hydrogen and hydrosulphuret of ammonia; but the precipitate in both cases is redissolved by an excess of the last-mentioned reagent." (p. 505.)

As a test for oxalic acid, when in a pure state, the addition of chloride of gold with the subsequent application of warmth to the liquids is recommended. The gold, as it is well known, is under these circumstances reduced and precipitated in a metallic state. (p. 522.)

It is commonly imagined that free sulphuric acid is one of the easiest of the poisons to detect; but it is proper to observe that there are some difficulties connected with this subject, which must not be overlooked. For example, any acid, vegetable, or mineral, mixed with a solution of a common sulphate, might be erroneously pronounced to be sulphuric acid. Dr. Christison has fully pointed out this source of error; but the means which he recommends for the avoidance of it, are somewhat complicated. It may happen that sulphuric acid may be mixed with a sulphate in a liquid submitted to analysis. In such a case, carbonate of barytes may be added to the liquid warmed, and the whole of the free sulphuric acid will thus be thrown down as sulphate of barytes. Dr. Simon's method of separation is as follows:

"As free sulphuric acid is soluble in pure alcohol, and the sulphates are insoluble in that menstruum, the liquid should, after filtration, be evaporated at a gentle heat to a small bulk, and then digested with absolute alcohol. This alcoholic solution should be set aside for twenty-four hours, again evaporated to a syrupy consistency, and the residue again treated with pure alcohol. On filtration, the alcohol may be separated from the sulphuric acid mixed with it by evaporation, and the acid then discovered by any barytic salt." (p. 527.)

This appears to us to be a better process than any yet suggested by toxicologists for separating free sulphuric acid from mixed sulphates. Dr. Simon makes no further application of it; but we think it might be made serviceable for the separation of any acid capable of combining with alcohol, (as the acetic) from sulphates, and render unnecessary the process of distillation recommended by Dr. Christison.

The process for hydrocyanic acid is not very satisfactory, at which we must express our surprise, considering the great simplicity of the analysis. Nitrate of silver is recommended as a test; but it is said that the cyanide of silver in drying becomes almost black, a statement opposed to the most common observation; for it is one of the least changeable of the salts of silver, and this has been assigned to it as one of its chemical characters by Mr. Barry. We agree in the statement that it differs from the chloride, in being easily decomposed by heat; but the author does not say that when heated it yields cyanogen, which burns with a peculiar flame, and thus renders the salt easily distinguishable from every other compound of silver. (p. 553.)

To prevent the loss of this poison in separating it by distillation from any organic mixture it is recommended to place a small quantity of alcohol in the receiver, with a few drops of caustic potash. It is customary to employ, under these circumstances, nitrate of silver, a solution of which is placed in the receiver; but, as Dr. Simon observes, it is possible that muriatic acid may be present and be distilled over, thus giving rise to ambiguity. To prevent this, the free acid in the liquid should be first neutralized by potash, and then, before distillation, it may be slightly acidulated with acetic (tartaric) acid. (p. 537.)

The author prefers, however, to use the ammonio-nitrate of silver in the receiver, by which he thinks the vapour of the poison is more likely to be absorbed; diluted nitric acid will afterwards throw down the cyanide. He omits to mention a very important chemical distinction between the cyanide and chloride of silver, namely, that the former is entirely soluble in caustic potash, while the latter is insoluble. He recommends as an additional test, the addition of sesquichloride of iron to the solution of cyanide in ammonia, in order to produce Prussian blue. The production of Prussian blue from the solution of cyanide of silver in potash, is an excellent addition to the tests for prussic acid, and has for some time been known in this country. It is not, however, noticed in works on toxicology. The author evidently imagines, that this modification of applying two good tests to one single portion of suspected liquid is original; but with the exception of the substitution of ammonia for potash as the solvent, by which no advantage is gained, it has been long practised here.

The chapter on opium is defective, both pathologically and chemically. The symptoms are dismissed in five lines, and the post-mortem appearances in two. Indeed, if we except the chemical analysis of poisons, we must look upon the author's mode of treating toxicology rather as furnishing a synopsis than a finished essay of the subject. The doses of the poisons required to prove fatal, the time within which death takes place, the antidotal treatment, and numerous other interesting questions relative to the practical part of the subject, are not even referred to.

It is not common to find the chemical processes for detecting strychnia and brucia in works on toxicology; and we shall, therefore, extract a few of Dr. Simon's remarks on this difficult subject. Some years since, it was scarcely possible to obtain a specimen of strychnia which did not contain traces of brucia; hence it was difficult to distinguish these two bodies from each other, which otherwise bear a close resemblance. Strychnia may now, however, be procured quite pure, and the following are the tests for its presence:

"Concentrated nitric acid dropped upon strychnia or its salts gives to it a lemon-yellow colour; if the strychnia contain any brucia, the colour is first of a light rose, passing afterwards into a deep red. Brucia is first changed by nitric acid to a rose red, and afterwards to a deep blood-red tint. A solution containing 1-100th of strychnia, is scarcely affected by concentrated nitric acid; if any brucia be present, an orange colour is produced. A solution of brucia, of equal strength with that just mentioned, is turned by a few drops of strong nitric acid, to a dark Malaga wine colour. Pure strychnia, treated in the same way with concentrated sulphuric acid, acquires a yellowish colour; if it contain brucia, it is at first rose-red, and afterwards of a brown-red. Pure brucia becomes of a deep brown-red, and finally passes to an olive-green. Sulpho-cyanate of potash causes in a solution of strychnia containing 1-100th, a turbidness soon followed by a white precipitate. If less concentrated, fine needle-shaped crystals form in the liquid after some time, which gradually increase in size. In a similar solution of brucia, no turbidness or precipitate occurs on adding the sulphocyanate, and it is only after some hours that a horny crystalline deposit attaches itself to the sides of the vessel. Both solutions are precipitated by infusion of galls, or any liquid containing tannin." (p. 546.)

As a poison, it is necessary to remember that impure strychnia is much more likely to be seen than that which is absolutely pure. We have seldom met with a specimen of strychnia which has not been turned of a red or orange colour by nitric acid; and this colour in the course of an hour commonly passes to a dark brownish green, a character which strikingly distinguishes it from morphia similarly treated. This is at first of a rich red, and slowly passes by exposure to a light yellow tint.

In order to separate strychnia or brucia from the contents of the stomach, the following process may be pursued:

"We must separate the solid from the liquid portions, digest the solid parts repeatedly in acetic acid, then mix the resulting liquid, and evaporate to dryness in a water-bath. This residue must be treated with alcohol, and to the concentrated alcoholic liquid, ammonia may be added to precipitate the alkaloids. The precipitate should be redissolved in acetic acid, and decolorized by boiling with animal charcoal. On evaporating this mixture, and digesting in alcohol, the acetate of strychnia or brucia may be obtained in a crystalline form, by spontaneous evaporation, or, as it sometimes happens, a very bitter extract remains, which is affected, like strychnia or brucia, by the addition of nitric acid." (p. 547.)

Perhaps this is as simple a method of obtaining these alkalies from the stomach in cases of poisoning, as any that can be suggested; but it will be seen that there is a good deal of difficulty and trouble attending the process; nor can the operator always hope to succeed unless he has had some experience in extracting these alkaloids from the substances in which they exist.

With regard to nux vomica, this can only be detected by seeking for the strychnia which it contains, having first separated as much as possible all foreign matters from the residue of the poison found in the stomach. In this case, we must boil the substance repeatedly in alcohol of 60 or 70 per cent., filter, concentrate and boil this alcohol extract, with calcined magnesia and water. This causes a precipitation of the strychnia, which may be afterwards separated from any extraneous matter by boiling alcohol. (p. 545.)

We here conclude our notice of Dr. Nicolai's work. Our remarks on chemical toxicology have been full; because it is now some years since a new edition of Christison's treatise has appeared; and there is no other English work on the subject that we can recommend. In the mean time, many improvements have been made in the processes for detecting poisons. We consider this, on the whole, to be a useful manual of medical jurisprudence. For the benefit of those, who wish to study the subject more deeply, the author has attached to each section the names of the authorities whom it would be advisable to consult. A work of this description is much wanted in the English language.

